# Vascular disrupting action of electroporation and electrochemotherapy with bleomycin in murine sarcoma

**DOI:** 10.1038/sj.bjc.6604168

**Published:** 2008-01-08

**Authors:** G Sersa, T Jarm, T Kotnik, A Coer, M Podkrajsek, M Sentjurc, D Miklavcic, M Kadivec, S Kranjc, A Secerov, M Cemazar

**Affiliations:** 1 Institute of Oncology Ljubljana, Zaloska 2, SI-1000 Ljubljana, Slovenia; 2 Faculty of Electrical Engineering, University of Ljubljana, Trzaska 25, SI-1000 Ljubljana, Slovenia; 3 Faculty of Medicine, University of Ljubljana, Korytkova 2, SI-1000 Ljubljana, Slovenia; 4 Institute Jozef Stefan, Jamova 39, SI-1000 Ljubljana, Slovenia

**Keywords:** vascular disruption, electrochemotherapy, electroporation, bleomycin

## Abstract

Electrochemotherapy has a direct cytotoxic effect on tumour cells, and presumably, a vascular disrupting effect. In this study, on the basis of the prediction of the mathematical model, histological evaluation and physiological measurements of the tumours were carried out to confirm that electroporation and electrochemotherapy of tumours have a vascular disrupting action. In the study, SA-1 solid subcutaneous sarcoma tumours in A/J mice were treated by bleomycin (BLM) given intravenously (1 mg kg^−1^), application of electric pulses (8 pulses, 1040 V, 100 *μ*s, 1 Hz) or a combination of both – electrochemotherapy. The vascular effect was determined by laser Doppler flowmetry, power Doppler ultrasonographic imaging and Patent blue staining. The extent of tumour hypoxia was determined immunohistochemically by hypoxia marker pimonidazole and partial pressure of oxygen (pO_2_) in tumours by electron paramagnetic resonance oximetry. Electrochemotherapy with BLM induced good antitumour effect with 22 days, tumour growth delay and 38% tumour cures. The application of electric pulses to the tumours induced instant but transient tumour blood flow reduction (for 70%) that was recovered in 24 h. During this tumour blood flow reduction, we determined an increase in hypoxic tumour area for up to 30%, which was also reflected in reduced tumour oxygenation (for 70%). According to the described mathematical model, endothelial cells lining in tumour blood vessels are exposed to a ∼40% higher electric field than the surrounding tumour cells, and therefore easily electroporated, allowing access of high BLM concentration to the cytosol. Consequently, electrochemotherapy has, besides the immediate vascular disrupting action, also a delayed one (after 24 h), as a consequence of endothelial cell swelling and apoptosis demonstrated by extensive tumour necrosis, tumour hypoxia, prolonged reduction of tumour blood flow and significant tumour growth delay, and tumour cures. Our results demonstrate that in addition to the well-established direct cytotoxic effect on tumour cells, electrochemotherapy also has an indirect vascular disrupting action resulting altogether in extensive tumour cell necrosis leading to complete regression of tumours.

*In vivo* electroporation has found its place as a drug delivery system for chemotherapeutics, that is electrochemotherapy, and is currently under intensive investigation for delivery of therapeutic genes to normal tissues and tumours, that is gene electrotransfer (Neumann *et al*, 1982; Cemazar *et al*, 2006; Heller and Heller, 2006; Mir, 2006). Bleomycin (BLM) and cisplatin proved to be effective in electrochemotherapy for treatment of various tumours in mice, rats, cats, dogs and horses (Sersa *et al*, 2006b). In a clinical setting, several studies predominantly on patients with malignant melanoma, basal cell carcinoma, head and neck tumours, and others have proved antitumour effectiveness of electrochemotherapy by long-lasting local tumour control (Heller *et al*, 1999; Marty *et al*, 2006; Sersa, 2006a).

The predominant underlying mechanism of electrochemotherapy is electroporation of cells in the tissues, which facilitates access of poorly permeant and nonpermeant molecules, including cytotoxic drugs such as BLM and cisplatin, into the cells (Mir, 2006). In addition to electroporation of tumour cells, electric pulses were found to modify blood flow in normal tissues and in tumours (Ramirez *et al*, 1998; Sersa *et al*, 1998, 1999b; Engstrom *et al*, 2001; Gehl *et al*, 2002). This phenomenon was well documented by different techniques demonstrating reduced blood flow and oxygenation in the tumours (Sersa *et al*, 1999b, 2002). In addition, we demonstrated that electroporation of adherent endothelial monolayer induced disruption of actin filaments and microtubule network cytoskeleton as well as loss of cell–cell contact junctions, which altogether increased endothelial monolayer permeability (Kanthou *et al*, 2006). This suggests that in addition to the direct effect of electric pulses applied to the tumour, there may also be an indirect effect on tumour via the effect of electric pulses on tumour blood vessels (vascular disrupting effect). Furthermore, we have previously demonstrated that electrochemotherapy with either BLM or cisplatin also has a tumour blood flow modifying effect (Sersa *et al*, 1999a, 2002). In addition, we showed that cultured endothelial cells are very susceptible to electrochemotherapy with BLM and to the same extent as tumour cells in the case of electrochemotherapy with cisplatin (Cemazar *et al*, 2001). Therefore, by electroporation of endothelial cells in tumour blood vessels, these may also become susceptible to chemotherapeutics, leading to endothelial cell death, vessel occlusion and abrogated tumour blood flow, thus inducing a cascade of cell death in the surroundings of the occluded blood vessel. If so, then electrochemotherapy is also currently a member of the intensively investigated vascular disrupting therapies that cause a rapid and selective shutdown of established tumour vasculature and can lead to secondary tumour cell death (Tozer *et al*, 2001, 2005; Siemann *et al*, 2005).

In this study, we present a mathematical model of electric field distribution in tumour blood vessels, predicting vascular disrupting action of electrochemotherapy. The prediction of the model is supported by the data demonstrating reduced tumour blood flow after electroporation and electrochemotherapy with BLM as well as pronounced tumour hypoxia, reduced tumour oxygenation, endothelial cells swelling and apoptosis, and extensive tumour cell necrosis. The study demonstrates that in addition to the direct effect on tumour cells, electrochemotherapy also has a vascular disrupting effect.

## MATERIALS AND METHODS

### Mice and tumours

Murine fibrosarcoma SA-1 (The Jackson Laboratory, Bar Harbor, ME, USA) cells were used in the experiments. A/J mice of both sexes, purchased from the Institute of Pathology, Faculty of Medicine, University of Ljubljana, Slovenia, were used for this study. They were kept at a constant room temperature (22°C) with a natural day/night light cycle in a conventional animal colony with food and water *ad libitum*. Before the experiments, the mice were subjected to an adaptation period of at least 10 days. The mice were 10–14 weeks old at the beginning of the experiments, weighing 20–25 g.

Tumour cells were obtained from the ascitic form of the tumours in mice. Solid subcutaneous tumours, located dorsolaterally in mice, were initiated by an injection of 5 × 10^5^ SA-1 cells in 0.1 ml 0.9% NaCl solution. Six to 8 days after implantation, when tumours reached approximately 40 mm^3^ in volume, the mice were randomly divided into experimental groups. The study was approved by the Ministry of Agriculture, Forestry and Food of the Republic of Slovenia (No. 323-02-237/01) in compliance with the Guide for the Care and Use of Laboratory Animals (National Institutes of Health, Bethesda, MD, USA).

### Electrochemotherapy

Bleomycin (Heinrich Mack Nachf., Illertissen, Germany) at a dose of 1 mg kg^−1^ (∼20 *μ*g per mouse) was injected intravenously. Injection volume was 7.5 ml per kg body weight. Eight square wave electric pulses in two sets of four pulses, in two mutually perpendicular directions of 1040 V (at voltage to distance ratio 1300 V cm^−1^) and with a pulse duration of 100 *μ*s and repetition frequency of 1 Hz, were delivered by two flat, parallel stainless-steel electrodes 8 mm apart (two stainless-steel strips: length 15 mm, width 7 mm, with rounded corners), which were placed percutaneously at the opposite margins of the tumour. Good contact between the electrodes and the skin was assured by means of a conductive gel. Electric pulses were generated by a Jouan GHT 1287 electroporator (Jouan, Saint Herblain, France). In the electrochemotherapy protocol, the mice were treated with electric pulses 3 min after BLM injection. All treatments except in the case of electron paramagnetic resonance (EPR) oximetry, laser Doppler measurements and power Doppler ultrasonographic imaging (see below), were performed without anaesthesia and were well tolerated by the animals.

Tumour growth was followed by measuring three mutually orthogonal tumour diameters (*e*_1_, *e*_2_ and *e*_3_) with a Vernier calliper three times per week. Tumour volumes were calculated according to the formula for the volume of an ellipsoid, *V*=*πe*_1_*e*_2_*e*_3_ × 6^−1^. The AM (arithmetic mean) of tumour volumes and s.e. were calculated for each experimental group. Tumour doubling time was determined for each individual tumour from the growth curves. Tumour growth delay was calculated for each individual tumour by subtracting the doubling time of each tumour from the mean doubling time of the control group and then averaged for each experimental group. Animals that were tumour free for 100 days were declared cured.

### Histology

At different time points after treatment with BLM, the application of electric pulses and electrochemotherapy of tumour's morphological changes, extent of tumour necrosis and the area with hypoxia marker pimonidazole adduct staining were determined. Tumour cell and blood vessels morphology as well as the extent of tumour necrosis were estimated on haematoxylin and eosin-stained slides. For necrotic tumour area analysis, the slides of three tumours per group were examined by two co-authors blinded to therapy status. The area of tumour necrosis was determined in a whole mount tumour section through the largest tumour diameter and expressed as a percentage of necrosis in relation to the whole tumour cross-section area. If the difference between two observers were greater than 10%, a consensus was achieved.

The hypoxia marker pimonidazole (Hypoxyprobe™-1, Natural Pharmacia International Inc., Belmont, MA, USA) (60 mg kg^−1^ in 0.2 ml PBS) was injected intraperitoneally. Tumours (3–9 per group) were excised 90 min thereafter, fixed in 10% buffered neutral formalin, embedded in paraffin, cut at three different levels throughout the tumour and stained for pimonidazole according to the manufacturer's instructions. Briefly, the sections were deparaffinised with xylene and rehydrated in ethanol. Sections were exposed to 3% H_2_O_2_ in methanol for 5 min to quench endogenous peroxidase activity. Antigen retrieval was achieved by incubating tissue sections with 0.01% pronase for 25 min at 40°C. Sections were then incubated with 1 : 50 dilution of monoclonal anti-pimonidazole antibody (Natural Pharmacia International Inc.) for 40 min at room temperature. This step was followed by biotin-conjugated rabbit anti-mouse secondary antibody and peroxidase-conjugated avidin–biotin complex (Dako, Glostrup, Denmark). Finally, sections were incubated with 3,3′-diaminobenzidine peroxidase substrate and stained with haematoxylin. The slide in which primary antibody was omitted served as a negative control. The mean percentage of area positive for pimonidazole adducts was determined by the image-based analysis system LUCIA (Nikon, Tokyo, Japan). The results of the percentages of necrotic tumour area and the percentages of pimonidazole-positive area were presented as AM and s.e. for each experimental group.

### Mathematical model of electric field distribution in and around a blood vessel

The lumen of a tumour blood vessel was modelled as a cylinder with a diameter of 15 and 8 *μ*m and an electric conductivity of 0.75 S m^−1^ for larger tumour vessels and 0.6 S m^−1^ for tumour capillaries, which is the electric conductivity of the blood serum (Hirsch *et al*, 1950, Tozer *et al*, 2001). The wall of the blood vessel was modelled as a layer with a thickness of 1 *μ*m and the tumour tissue as a homogeneous material surrounding the vessel wall. Both regions were assigned an electric conductivity of 0.3 S m^−1^, which is an average value of reported bulk electric conductivities of soft tissues (including epithelial and tumour tissues) that range from 0.1 to 0.5 S m^−1^ (Geddes and Barker, 1967; Duck, 1990; Gabriel *et al*, 1996). The distribution of the electric potential was derived analytically by solving the Laplace equation in cylindrical coordinates with the boundary conditions of homogeneity of the electric field far from the vessel, boundedness and continuity of the electric potential, and continuity of the electric current density (Kotnik *et al*, 1997; Kotnik and Miklavcic, 2000). The distribution of the electric field was then determined by calculating the gradient of the electric potential.

### Power Doppler ultrasonographic imaging

The gross tumour perfusion changes after application of electric pulses, BLM and electrochemotherapy to the tumours were evaluated by power Doppler ultrasonographic imaging. Before and after (at 3 min, 35 min and 48 h) different therapies, grey-scale ultrasound (US) of tumours was performed. The longest and the shortest diameter of tumour and the longest diameter of an echogenic central part (necrosis) were measured. After baseline, US scan evaluation power Doppler US was performed at each time point to check vascularisation. Before microbubble contrast agent (Sono Vue, Bracco, Milan, Italy) injection, the persistence of the image display on the US machine was set to zero, the signal gain was registered below the noise threshold, and one focus was positioned below the level of the lesion. SonoVue is a sulphur hexafluoride-filled microbubble contrast agent that is licensed for use in abdominal and vascular imaging in most European countries. This agent has a strong nonlinear harmonic response when it is insonated with low acoustic power. The safety and effectiveness of this agent in vascular and parenchymal diagnostic applications have been proved in preliminary experimental and clinical investigations (Delorme and Krix, 2006).

SonoVue (5 mg ml^−1^) was mixed with a solution of sodium chloride 0.9% w v^−1^ solution to form a dispersion containing millions of tiny bubbles of sulphur hexafluoride gas. The solution was injected intravenously (0.2 ml) in mice by using a 27-gauge needle. After 5–10 s, we observed strong peripheral enhancement, and central necrosis was better depicted as on baseline US image. If contrast enhancement was considered inadequate by the on-site examinator after the first bolus injection, the mouse received an additional microbubble contrast agent bolus (0.2 ml). We monitored contrast enhancement for at least 5 min after injection. During the measurements, the mice were anaesthetised in the same way as for EPR oximetry measurements. All US examinations were performed by one experienced radiologist on a US machine (Aplio HU 80; Toshiba, Otawara, Japan) with a 12 MHz, 38-mm long linear transducer. The scanning plane was selected for optimal visualisation of enhancement. Dynamic US data were stored in the US unit until the signal enhancement had completely diminished, usually within 5 min. Static images were obtained before and every 15 s after injection of the contrast agent. The whole examination was stored on a CD for further analysis.

### Laser Doppler flowmetry

Relative blood perfusion was monitored using an OxyFlo2000 Laser Doppler flowmeter and OxyData data acquisition unit (Oxford Optronix Ltd, Oxford, UK). The signals were sampled and stored at a frequency of 100 Hz. The theory and applications of laser Doppler flowmetry (LDF) are well known (Shepherd and Oberg, 1990); briefly, LDF measures the spread of the wavelengths of photons emitted by a coherent laser source when the photons scatter on moving red blood cells in capillaries. The distribution of photon wavelengths is used for calculation of relative microcirculation in tissue.

For skin measurements, LDF can be used entirely noninvasively. In our study, we, however, used thin invasive probes (diameter 200 *μ*m) to assess the perfusion inside the tumour (Jarm *et al*, 2002). To minimise discomfort of the mice and to keep them as motionless as possible during 1 h continuous monitoring, the mice were anaesthetised using isoflurane (flurane-isoflurane; Abbott Labs, Berkshire, UK). Isoflurane was delivered to the mice via a miniature face mask in a mixture of oxygen and nitrous oxide (flow of each 0.6 l min^−1^) at 3.0 and 1.8% concentrations, respectively, for induction and maintenance of anaesthesia. Although anaesthetised, the mice were kept on an automatically regulated heating pad to prevent hypothermia. Rectal temperature was kept as close as possible to 37°C with variations of up to 0.5°C during single measurements and with the contact surface temperature of the heating pad always below 39°C.

Approximately 4 min after the induction of anaesthesia, the data acquisition was started and a probe inserted in the tumour. The probe was inserted through a small superficial incision in the skin, pushed a few millimetres into the tumour and then slightly withdrawn to minimise the pressure of the tip of the probe on the surrounding tissue. After approximately 30 min, either BLM or physiological saline was injected intravenously. Exactly 3 min after the injection, the electrodes were attached to the tumour and the electric pulses delivered. The electrodes were then removed and the blood flow signal recording continued for at least 1 h afterwards. In the control and BLM groups, the electrodes were manipulated in the same way as in the groups treated by electric pulses or electrochemotherapy, except that no electric pulses were applied. Special care was taken throughout the measurements to minimise all movements of the probes and the mouse to keep the movement artefacts in recorded signals as small as possible.

After the measurement, the raw laser Doppler signals were first filtered using a special custom-made digital filter to remove movement artefacts caused mostly by respiration of the mice (see Figure 5A as an example), thus resulting in a smoothed and downsampled (1 Hz) version of the raw signal, which represented the actual baseline blood flow level. We defined the moment 1 sec before the application of the first electric pulse (3 min after injection) as time zero (see Figure 5B). From the filtered signal, average blood flow values at distinct predefined time moments before, at and after the treatment were calculated. In general, these time moments were spaced at intervals of 5 min except around time zero, where they were distributed more densely to track rapid changes in baseline blood flow levels occurring after the treatment. Average blood flow values were calculated from filtered signal segments of 20 s length cantered at the aforementioned time moments. As the last step, all average blood flow values were normalised with respect to the average blood flow level measured 5 min before time zero (pretreatment value at −5 min in Figure 5B and C). Thus, the blood flow level in each tumour was expressed as a percentage of the pretreatment blood flow level. These relative values were then used to present the final results and to evaluate the effects of different treatments on tumour blood flow.

### Assessment of tumour perfusion by Patent blue staining

Patent blue (Byk Gulden, Krenzlingen, Switzerland) was used to estimate tumour perfusion. Patent blue (1.25%) diluted in 0.2 ml 0.9% NaCl was injected at different time points after treatment into the tail vein of animals with tumours from the control, electric pulses, BLM and electrochemotherapy groups. After the dye was distributed evenly through the tissue (1 min), animals were killed and the tumours carefully excised. The excised tumours were removed from the skin, cut in half along their largest diameter and photographed. The images were evaluated using Image J software. The stained area of tumour cross-section was used as an indicator of tumour perfusion. In the study comparing Patent blue staining and the pharmacological method measuring the relative tumour blood flow, a good correlation (*r*=0.962) was found between both methods (Sersa *et al*, 1999a, 1999b).

### Electron paramagnetic resonance oximetry

Electron paramagnetic resonance (EPR) oximetry was used to evaluate changes in the partial pressure of oxygen (pO_2_) in tumours and subcutaneous tissue on the contralateral side before and after treatment of tumours with electric pulses, BLM and electrochemotherapy. Electron paramagnetic resonance oximetry is a non-invasive method (after insertion of the paramagnetic probe), which allows monitoring of pO_2_ repeatedly at the same point in the tissue over long periods of time (O'Hara *et al*, 2001; Sersa *et al*, 2002). For this purpose, a paramagnetic probe, a char (∼0.5 mm^3^, with particle size of approximately 10 *μ*m) from Bubinga tree (a kind gift of EPR Center for Viable System, Dartmouth College of Medicine, Hanover, NH, USA), which is sensitive to oxygen, was implanted into the tumour centre and periphery (tumour volume approximately 40 mm^3^) and in the subcutis 1 day before the treatments. Its EPR spectral linewidth was measured at different time points up to 2 days after the treatment. The char implantation with a sterile needle did not provoke bleeding. In the presence of oxygen, the linewidth of the EPR lines is broadened; the extent of broadening depends on pO_2_ (Swartz *et al*, 1993; Swartz and Clarkson, 1998). The pO_2_ in the region in contact with the probe was measured.

The measurements were performed on a Varian E-9 EPR spectrometer with a custom-made low-frequency microwave bridge operating at 1.1 GHz and an extended loop resonator (11 mm in diameter), both designed by Professor T Walczak (Dartmouth College of Medicine, Hanover, NH, USA). Typical spectrometer settings were modulation frequency, 100 kHz; modulation amplitude not more than one-third of the peak-to-peak linewidth, and scan range, 2 mT. The linewidth of the EPR spectra reflects the pO_2_, which was determined from an existing calibration curve (Krzic *et al*, 2001).

For EPR oximetry, animals were anaesthetised with a mixture of Domitor (1.0 mg per kg body weight; Pfizer GmbH, Karlsruhe, Germany) and 10% ketamine (75.0 mg per kg body weight; Veyx-Pharma GmbH, Schwarzenborn, Germany) administered intraperitoneally. During anaesthesia, body temperature was kept at physiological values by a regulated heating pad on which the mice were placed (Guymar T/pump; Linton Instruments, Norfolk, UK).

### Statistical analysis

Significance tests were carried out on the data groups using analysis of variance followed by the Bonferroni *t*-test for individual pairwise comparisons, with values of *P*<0.05 considered as significant. The SigmaStat statistical software was used for statistical analysis (SPSS Inc., Chicago, IL, USA).

## RESULTS

### Antitumour effect of electrochemotherapy

Electrochemotherapy with BLM was very effective in treatment of subcutaneous SA-1 tumours, resulting in substantial tumour growth delay and high percentage of tumour cures, compared to untreated tumours and tumours treated with BLM or application of electric pulses alone (Table 1).

### Tumour histology after electrochemotherapy

**Figure 1:**
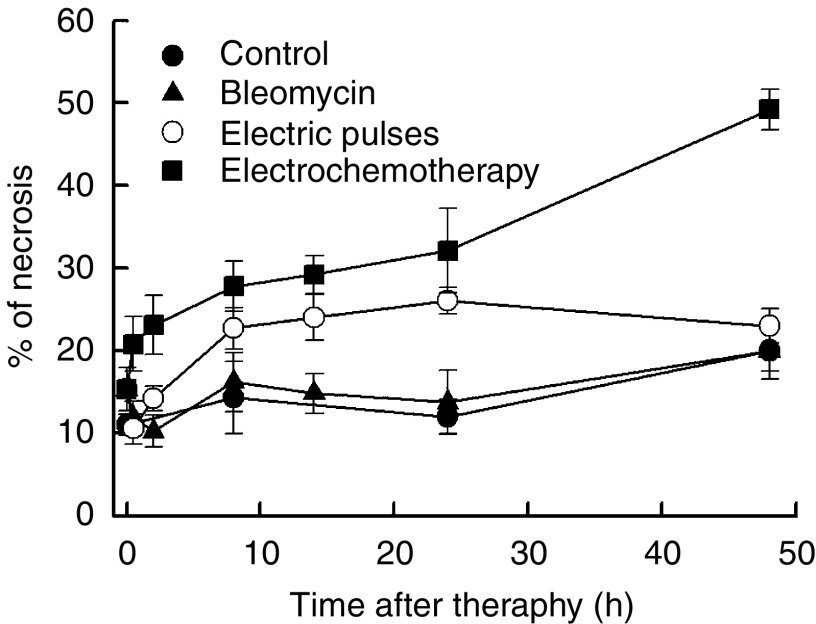
Time course of changes in tumour necrosis in untreated control tumours, and after treatment of tumours with bleomycin, application of electric pulses or electrochemotherapy. Symbols indicate AM (arithmetic mean)±s.e. of at least three mice per point.

Electrochemotherapy with BLM had, as already mentioned, significant antitumour effect on tumour growth. We specifically observed significant increase in extent in tumour necrosis reaching ∼50% at 48 h after the treatment (*P*<0.05 compared to other treatment groups). The necrotic areas were found mostly in the centre of the tumours and consisted of cells with eosinophilic cytoplasm and pycnotic nuclei. In some cells, acute cellular swelling and nuclear disappearance with cell ghosts were also found. Central necrosis was surrounded by a thin viable tumour rim that led to regrowth after 8–10 days in those tumours that were not cured (Figure 1). The extent of tumour necrosis in the tumours treated with BLM only was the same as in the untreated tumours, confirming the data on tumour growth. The application of electric pulses alone increased the extent of necrosis in the tumours, but only transiently, returning to the normal values within 48 h after treatment. This small but significant increase (*P*<0.05) in the extent of tumour necrosis up to 24 h after the treatment confirmed the previous observation that the application of electric pulses alone to the tumours induced a small but insignificant retardation in tumour growth (tumour growth delay=0.6 days, *P*>0.05).

**Figure 2:**
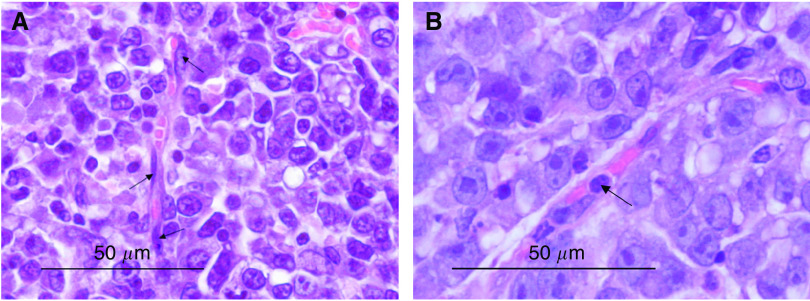
(**A**) Rounding up of tumour blood vessels endothelial cells 1 h after electric pulses increases vascular resistance to blood flow (arrows). (**B**) Apoptotic-like endothelial cells occur in tumours 8 h after electrochemotherapy (arrows).

Tumour slides were also examined for blood vessels changes. Changes in the endothelial cell shape were observed 1 h after the application of electric pulses. Endothelial cells were rounded up and swollen and the lumen of blood vessels was narrowed (Figure 2A). After electrochemotherapy, the same morphological changes were found. At later times after electrochemotherapy (8 h), in some vessels endothelial cells with apoptotic morphological characteristics were found (Figure 2B). Furthermore, blood vessels were stacked with erythrocytes and extravasation of erythrocytes was also observed (Figure 2B). Apoptotic endothelial cells were not observed in tumours treated with electric pulses alone, BLM alone or in the control group.

### Mathematical model of electric field distribution in and around a blood vessel

**Figure 3:**
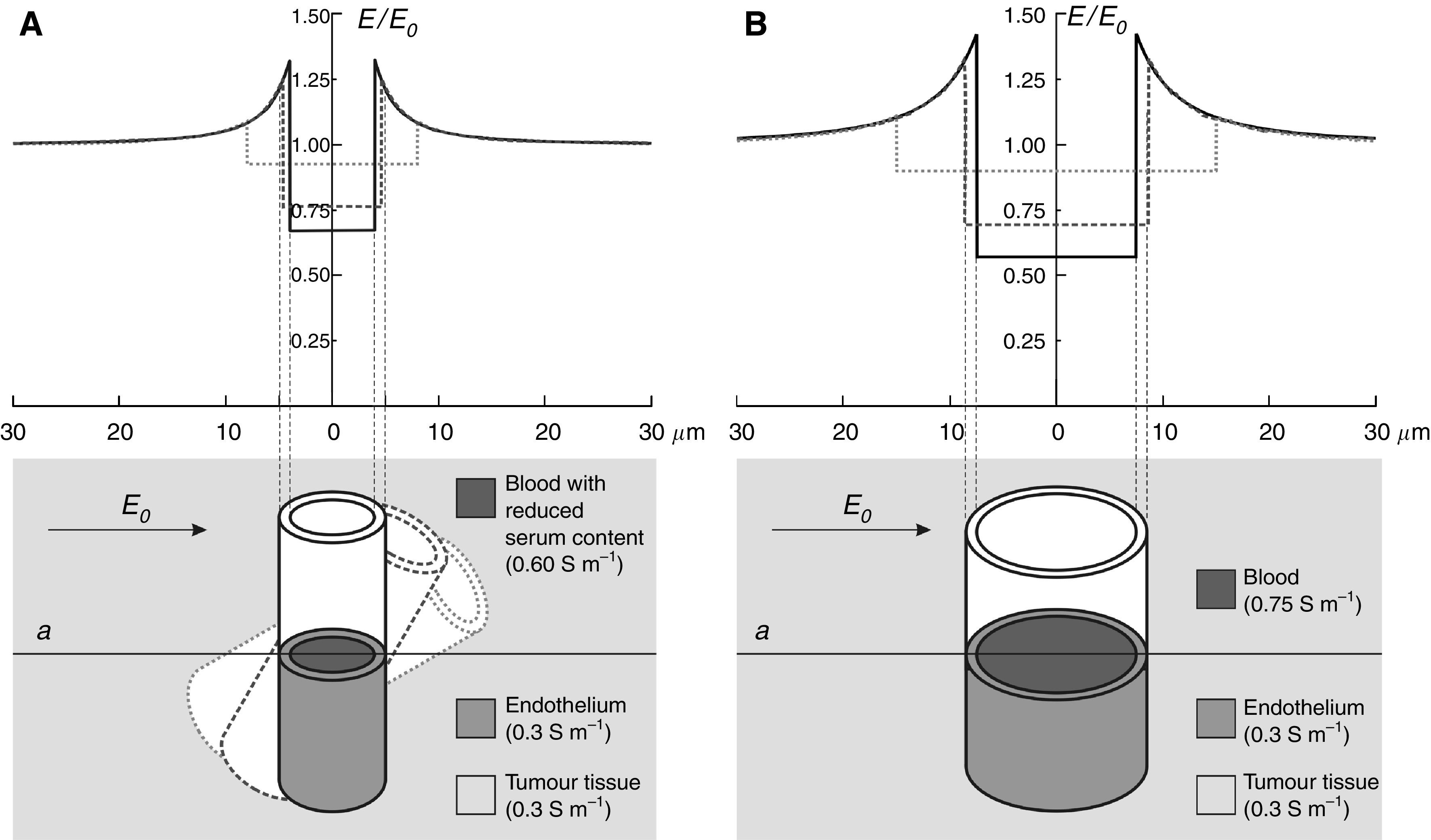
The electric field strength in a capillary of 8 *μ*m diameter (**A**), a vessel of 15 *μ*m diameter (**B**) and in the endothelial and tumour tissue surrounding them. The plot shows the ratio between the local electric field (*E*) along the line *a* and the external field to which the tissue is exposed (*E*_0_), for three different orientations of the vessel with respect to the field (90° – solid, 60° – dashed, 30° – dotted). The conductivities of the tissues and the blood are taken from the literature (Hirsch *et al*, 1950; Duck 1990; Gabriel *et al*, 1996). In the capillary, a somewhat lower serum content (and therefore conductivity) is assumed due to the erythrocytes filling most of the volume in the thin passages.

The local distortion of the electric field in the vicinity of a blood vessel is illustrated in the two plots in Figure 3, for a thin capillary of 8 *μ*m diameter (Figure 3A) and for a thicker tumour vessel of 15 *μ*m diameter (Figure 3B). Denoting by *E* the local field and by *E*_0_ the homogeneous field far from the vessel, the plots show the ratio *E*/*E*_0_ along the cross-section passing through the centre of the vessel (line *a*) for the vessel perpendicular to the field direction and for the vessel tilted by 30° and 60° with respect to the field direction. As the plots show, the electric field in the tissue at the very edge of the lumen, that is in the endothelium of the blood vessel, can exceed the homogeneous value by as much as 42% in the extreme case (the larger vessel, perpendicular orientation) and by 10–30% in typical cases, represented by different orientations of the vessels. This is the consequence of the significantly higher conductivity of the blood with respect to the surrounding tissue (see Materials and methods), due to which the electric current is locally concentrated in the vicinity and inside the blood vessel. In the blood, the electric field is only 60–90% of the homogeneous field in the tissue, which reflects the fact that the electric field is the ratio between the current density and the conductivity and that the conductivity of blood is considerably higher than the conductivity of the surrounding tissue. Consequently, the model predicts that endothelial cells lining the blood vessels are exposed to up to 40% higher electric field than the surrounding tumour cells.

### Tumour blood flow changes induced by application of electric pulses and electrochemotherapy

**Figure 4:**
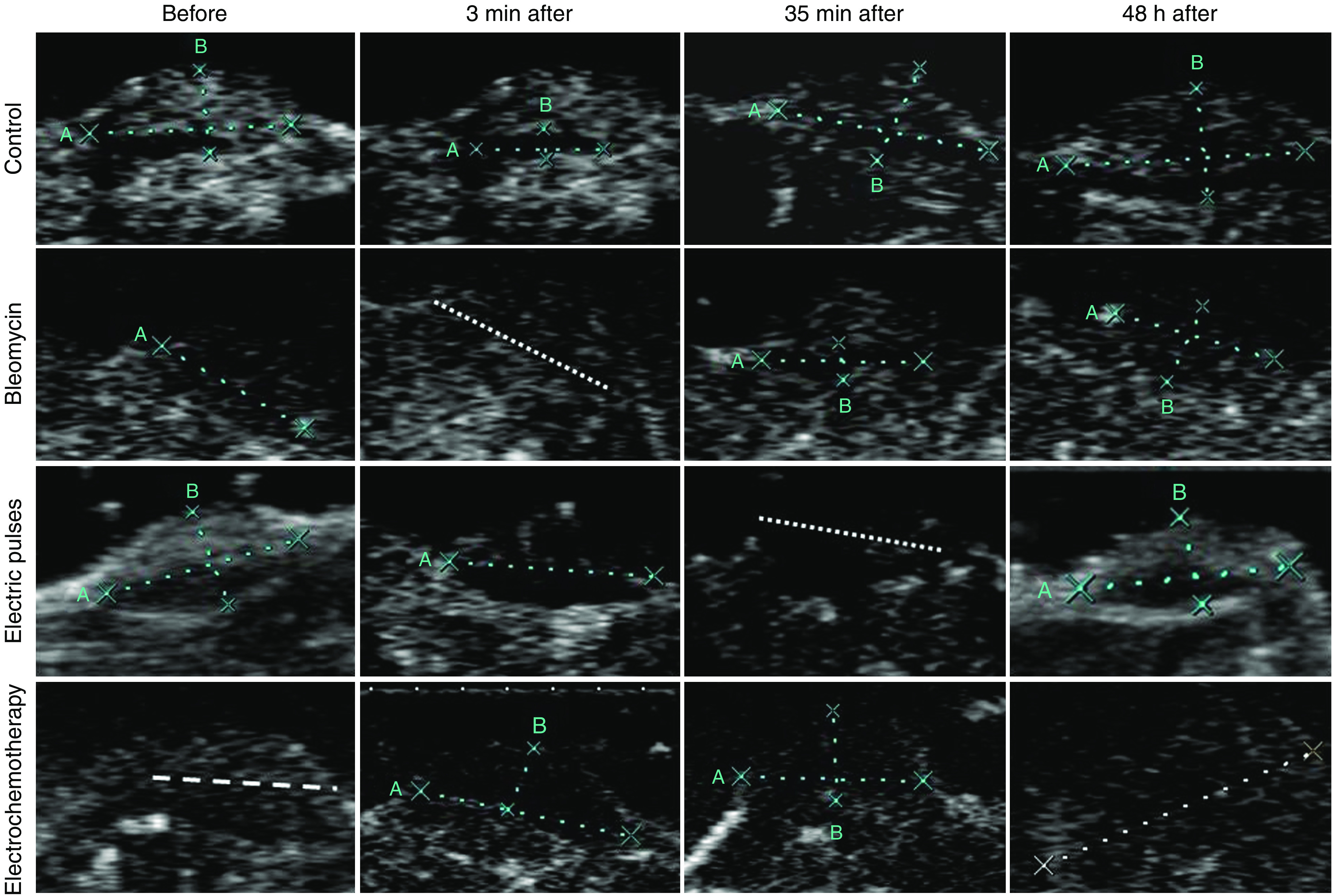
Representative power Doppler ultrasonographic images of tumours (delineated with white lines A and B) treated by bleomycin, electric pulses or electrochemotherapy at various time points post-treatment.

We have already demonstrated in previous studies that electric pulses and electrochemotherapy significantly reduce the blood flow of the treated tumours (Sersa *et al*, 1999a, 1999b). In this study, we used two new approaches to further evaluate this effect. We used power Doppler ultrasonographic imaging to assess the gross tumour blood flow changes and LDF to monitor local microvascular blood flow in the tissue. The application of eight electric pulses to the tumours reduced the blood flow of the tumours immediately after their application. Power Doppler ultrasonographic imaging showed immediate abrogation of blood flow in the vessels supplying the tumour, with restoration of blood flow within 48 h after application of electric pulses alone (Figure 4). The same time course of the effect was observed after electrochemotherapy of the tumours. However, at 48 h after electrochemotherapy, a reduced tumour blood flow was still observed after electrochemotherapy, contrary to electric pulses alone. Blood flow in tumours in the control group and in tumours treated with BLM only did not change during the observation period.

**Figure 5:**
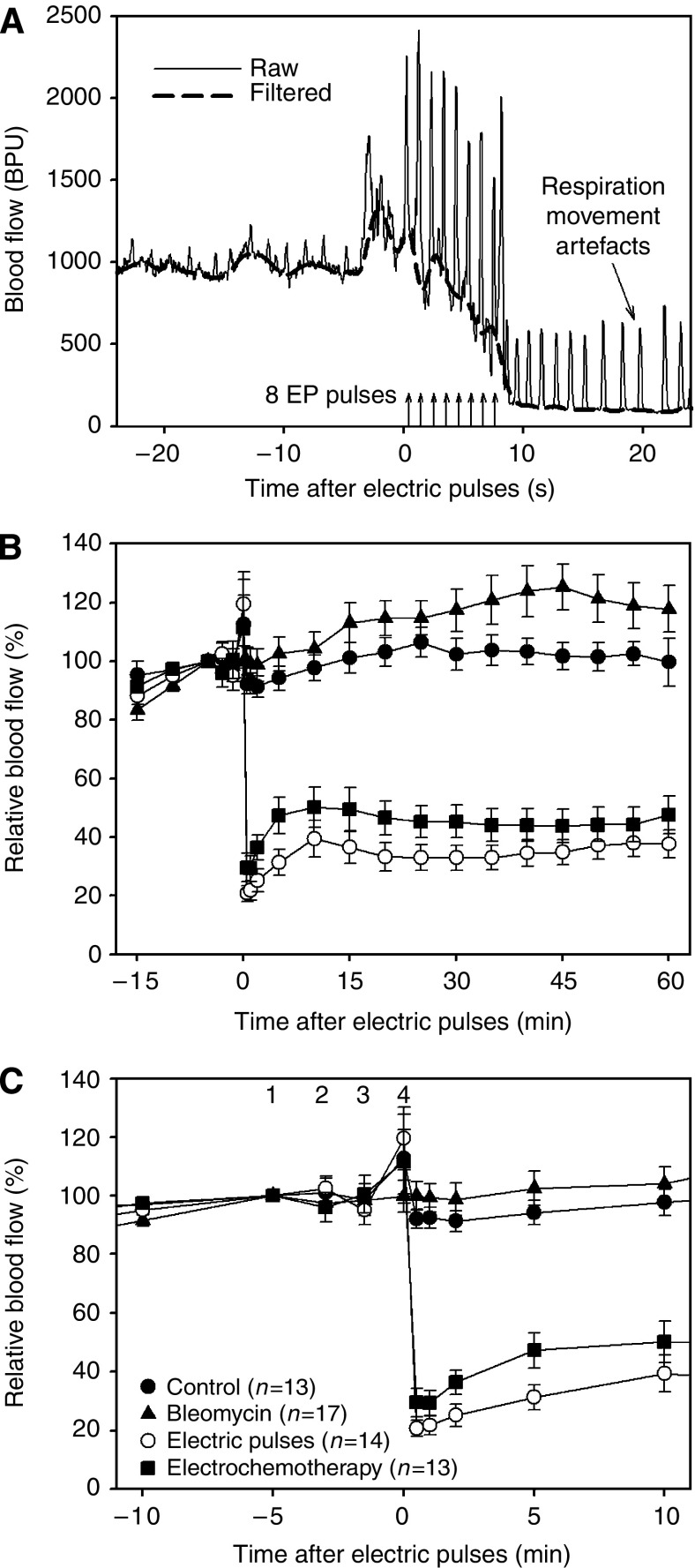
Blood flow changes after different treatments assessed by means of laser Doppler flowmetry. (**A**) Rapid decrease in blood flow immediately after application of electric pulses (EP). Movement artefact caused by the pulses, manipulation of the electrodes (spikes just before and after pulses) and respiration (before and after electroporation) can be observed in the raw signal along with the filtered signal. (**B**) Average blood flow values before and after treatment expressed as a percentage of the pretreatment blood flow (mean values with s.e. bars are shown). After time zero, the differences between the groups were statistically highly significant (*P*<0.001). (**C**) A close-up from (**B**): (1) pretreatment level at −5 min; (2) moment of injection at −3 min; (3) −1.5 min; (4) time zero. Relatively large scatter (nonsignificant) of values at time zero was of transient nature and was a result of movement artefacts caused by manipulation of the electrodes. Note different time scale.

We were further able to follow the exact profile of microcirculatory changes by means of LDF after application of electric pulses to the tumours or electrochemotherapy in a small volume of tumour tissue. Figure 5A shows a highly reproducible example of an extremely rapid decline in microcirculatory perfusion, which starts as soon as the first pulse is delivered and continues until the maximum reduction is obtained, which can occur as soon as 10 s after application of the first pulse. This figure also demonstrates various movement artefacts present in raw laser Doppler signal (owing to respiration, electrode manipulation and delivery of electric pulses) and thus the need for filtering of such a signal before any signal analysis.

Figure 5B and C show average changes in perfusion of tumours in the four experimental groups. The rapid and abrupt initial decrease in perfusion of tumours after application of electric pulses and electrochemotherapy-treated tumours was followed by a gradual but only partial reperfusion of the tumours. This partial reperfusion peaked approximately 10–15 min after delivery of electric pulses and did not continue afterwards. On the contrary, the injection of BLM or physiological saline (control) produced no significant changes during the observation period. The four experimental groups started to differ significantly as soon as 30 s after application of electric pulses and remained significantly different until the end of observation (*P*<0.001 for all time instants after time zero in Figure 5B). The differences were highly significant (*P*<0.001) between control and BLM alone compared to the group of tumours that was exposed to electric pulses or the electrochemotherapy group. Differences between the groups before and at time zero in Figure 5B were not statistically significant (*P*>0.05). It was also interesting to note that the two groups involving the use of BLM (BLM alone and electrochemotherapy) developed higher blood flow levels after treatment than the corresponding two groups without BLM (control and application of electric pulses, respectively), which can be observed in Figure 5B. However, even though this difference was consistent, it was not statistically significant (*P*>0.05).

**Figure 6:**
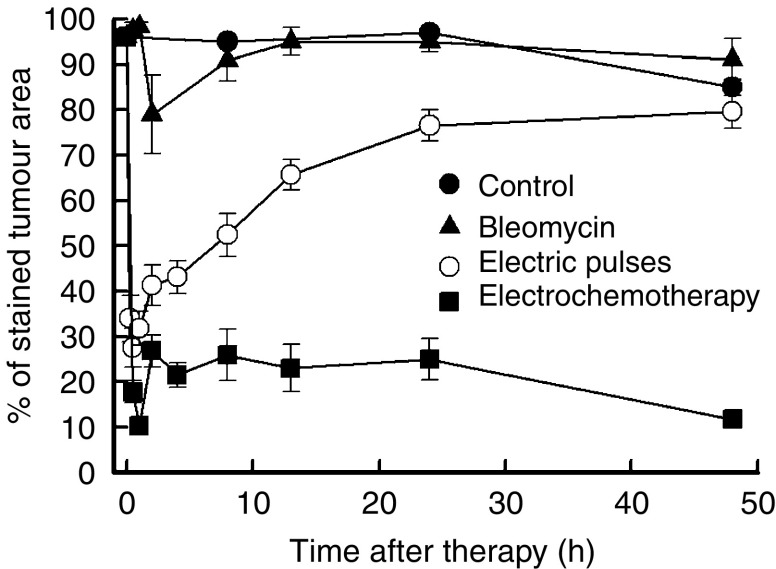
Time course of changes in tumour perfusion in untreated tumours, and after treatment of tumours with bleomycin, application of electric pulses or electrochemotherapy. Symbols indicate AM (arithmetic mean)±s.e. of at least three mice per point.

Tumour perfusion changes were also measured by Patent blue staining of the tumours with similar vascular effects as observed with other methods of measurement (Figure 6). An immediate reduction was observed in tumour perfusion after application of electric pulses and electrochemotherapy with reduction of tumour perfusion to 10% of the value in control tumours. Approximately 0.5 h later, the tumours started to reperfuse in both groups; in the tumours treated by electrochemotherapy, the reperfusion levelled at ∼1 h after the treatment and stayed at ∼20% up to 48 h after the treatment, whereas the tumours treated with application of electric pulses continued to reperfuse and reached normal values between 24 and 48 h.

### Tumour hypoxia induced by application of electric pulses and electrochemotherapy

Blood flow measurements showed that there is an immediate reduction of tumour blood flow after the application of electric pulses and electrochemotherapy. Owing to the effect of applied electric pulses on the vascular supply of the tumours, the extent of hypoxic regions in the tumours as a consequence of reduced tumour blood flow was determined.

**Figure 7:**
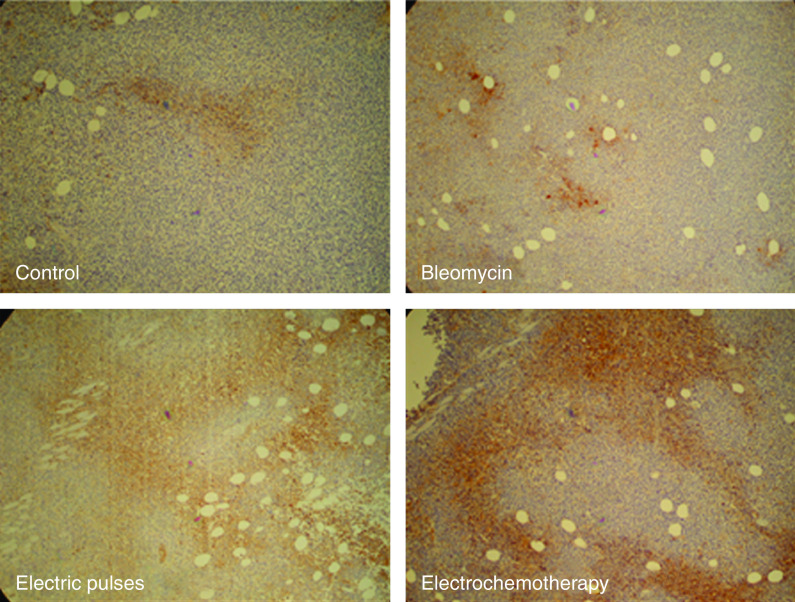
Representative tumour sections 90 min after treatment with bleomycin, application of electric pulses or electrochemotherapy. Brown regions are the cells stained with pimonidazole, a marker of tumour hypoxia. Note marked differences in the extent of hypoxic regions between groups without application of electric pulses (control and bleomycin) and those with the application of electric pulses (electric pulses and electrochemotherapy).

Tumour sections were immunohistochemically stained for protein adduct of reductively activated pimonidazole. Positive staining was found in the cytoplasm of tumour cells and some positive nuclei of tumour cells were also determined. As pimonidazole is known to be preferentially bound by hypoxic tumour cells, the detection of pimonidazole adducts using monoclonal antibodies can serve as a method for measuring tumour hypoxia. In most control tumours, discrete foci of pimonidazole positivity were found. In BLM-treated tumours, a rim of positive cells was found mainly around the necrotic area. In electric pulses and in the electrochemotherapy-treated group of tumours, reticular staining patterns were observed at some distance from blood vessels, with some diffuse pimonidazole-positive area. In some slides from the electrochemotherapy-treated group of tumours, the endothelial cells of blood vessels were also positive. The tumour cells around these pimonidazole-labelled vessels did not show pimonidazole immunoreactivity (Figure 7).

**Figure 8:**
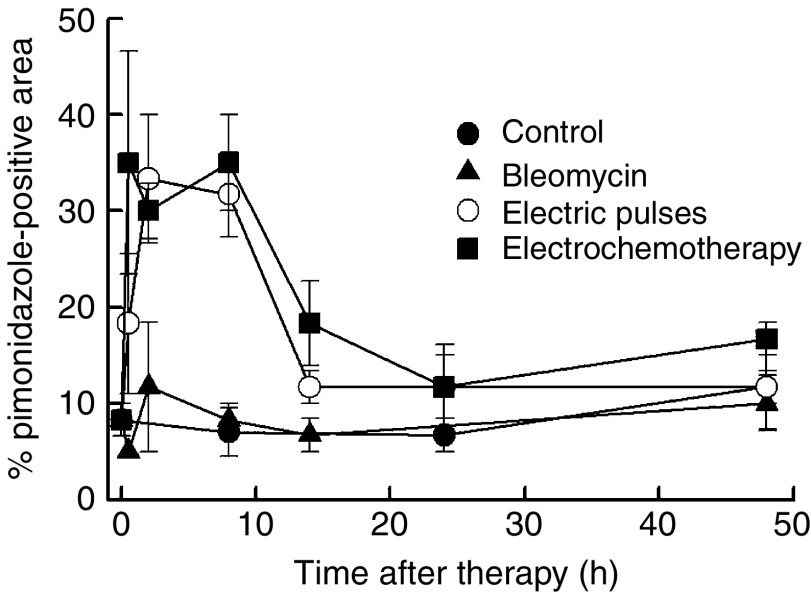
Time course of changes in tumour hypoxia in untreated control tumours, and after treatment of tumours with bleomycin, application of electric pulses alone or electrochemotherapy. Excised tumours were stained with pimonidazole, and percentage of pimonidazole positive tumour areas were determined. AM (arithmetic mean)±s.e. of at least three mice per point.

The percentage of pimonidazole-positive tumour areas in viable tumour tissue was determined and the results are presented in Figure 8. Both in the group of tumours treated with electric pulses and in those treated with electrochemotherapy, the onset of tumour hypoxia was instant, reaching its peak at 2 h and lasting up to 8 h after treatment. After the application of electric pulses, the recovery to the pretreatment level lasted 14 h, whereas the recovery to pretreatment level after electrochemotherapy lasted 24 h. From 24 to 48 h after treatment, the level of tumour hypoxia was the same in all experimental groups.

### Tissue oxygenation changes induced by application of electric pulses and electrochemotherapy

Partial pressure of oxygen was measured in animals treated with BLM and with the application of electric pulses to the tumours and electrochemotherapy, using EPR oximetry. In the same animals, the pO_2_ values were recorded in the tumours that were treated, as well as in the subcutaneous tissue that was not treated and was located on the contralateral side from the tumour. The pO_2_ values in the subcutaneous tissue were higher than those measured in the tumours. Furthermore, the pO_2_ values in the centre of the tumour were lower than those in the periphery of the tumours (Table 2).

**Figure 9:**
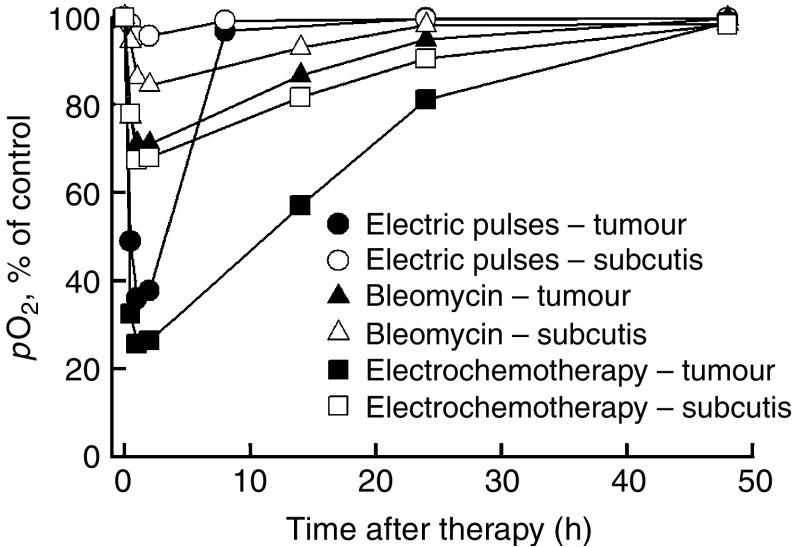
Time course of changes in partial pressure of oxygen (pO_2_) measured by EPR oximetry in tumours and subcutaneous tissue distant from the tumours, where electric pulses were not applied. The data were combined for tumour centre and tumour periphery. Animals were treated intravenously with bleomycin (1 mg kg^−1^) and 3 min later eight electric pulses were applied to the tumours (1040 V, 100 *μ*s, 1 Hz). Symbols indicate AM (arithmetic mean)±s.e. of six mice per point.

All treatments, even BLM alone, had a rapid effect on pO_2_ with maximal reduction of tissue oxygenation occurring within 2 h after the treatment (Table 2). We also followed the time course of pO_2_ changes relative to the pretreatment values of the specific treatment (Figure 9). The systemic injection of BLM induced some reduction in subcutaneous tissue (for 12%) and in tumour oxygenation (for 28%), with steady recovery to the pretreatment values within 48 h. The application of electric pulses to the tumours induced instantaneous reduction of pO_2_ to 38% of the pretreatment level, with steady recovery to pretreatment level within 8 h. Electrochemotherapy of tumours had a similar but more profound effect on the tumour oxygenation than the application of electric pulses alone. The onset and the degree of pO_2_ reduction in tumours after treatment with electrochemotherapy was immediate, and within 2 h it was same as with the application of electric pulses; however, the recovery of tumours the oxygenation was much slower, returning to the pretreatment level with a delay as long as 2 days. Application of electric pulses and electrochemotherapy also had some effect on oxygenation of subcutaneous tissue that was distant from the application of electric pulses. Electrochemotherapy in subcutaneous tissue induced bigger pO_2_ reduction (for 30%) than the application of electric pulses alone (for 8%).

## DISCUSSION

The results of our study demonstrate that besides its direct cytotoxic effect on tumour cells, electrochemotherapy with BLM also has a vascular disrupting action by causing a rapid shutdown of tumour blood flow, leading to reduced tumour oxygenation, increased tumour hypoxia and extensive tumour cell necrosis. Furthermore, owing to the blood vessel changes caused by a high concentration of BLM in the endothelial cells of tumour blood vessels, this therapy has prolonged vascular disrupting effects leading to pronounced antitumour effectiveness. The mathematical model of electric field distribution in and the around tumour blood vessel supports the physiological data by demonstrating that endothelial cells are exposed to a ∼40% higher electric field compared to the tumour tissue farther from the blood vessel.

Electroporation of tissues *in vivo* has great potential as a drug delivery system, as it is universal and easily applicable, also in humans. It can be used for the delivery of various molecules that have a hampered access to cytosol, such as some widely used chemotherapeutic drugs like BLM and cisplatin, or naked DNA (Cemazar *et al*, 2006; Sersa, 2006a). Several studies have indicated that electroporation of tissues, including tumours, induces intracellular accumulation of BLM or cisplatin, in the latter case also leading to an approximately twofold increase of binding of cisplatin to DNA (Belehradek *et al*, 1994; Cemazar *et al*, 1999). This enables increased cytotoxicity of the drugs such as BLM or cisplatin, particularly in the cells that are exposed to electric fields that cause their electroporation. Therefore, the antitumour effectiveness of electrochemotherapy was so far attributed predominantly to direct cytotoxicity of the delivered chemotherapeutic drugs to tumour cells, as a predominant population of cells in the tumours.

However, the application of electric pulses to the tissues, either muscles or tumours, induces vascular effects that need to be studied for possible interaction with other electroporation-based therapies such as gene electrotransfer. It has been shown already that the application of electric pulses to the tumours induces reduction of tumour blood flow that is profound but transient. The tumour vascular disrupting effects of electroporation were demonstrated both by rubidium extraction technique and Patent blue staining (Sersa *et al*, 1999a). This phenomenon was considered as beneficial, both for electrochemotherapy and gene electrotransfer. A consequence of reduced blood flow in the tissue is entrapment of the molecules delivered before electroporation of the tissue, providing longer exposure of the cells to the cytotoxic drugs, by preventing washout of the molecules from the tissue. Furthermore, in clinical cases it was observed that during electrochemotherapy, immediately after the application of electric pulses, the bleeding of haemorrhagic tumour nodules has stopped (Gehl and Geertsen, 2006). In our present study, we provide further evidence on previous observations about the tumour vascular disrupting action of electroporation. We demonstrated the instant abrogation of tumour blood flow after the applied electric pulses on the tumour, and recovery of tumour blood flow within 24 h. The instantaneous effect on blood flow was demonstrated by LDF and also by power Doppler ultrasonographic imaging as well as Patent blue staining. All approaches also demonstrated the recovery of tumour blood flow. Power Doppler ultrasonographic imaging using contrast agents is a noninvasive technique that is routinely used in a clinical setting and is therefore a very suitable method to assess the changes in tumour blood flow. In our study, we demonstrated the usefulness of this noninvasive technique for monitoring changes in blood flow in real time. Therefore, this technique could be used for planning of combined treatment approaches where antivascular therapies are combined with other therapies targeting hypoxic cells or therapies that should be applied at the time of maximal reperfusion after the first treatment (Shaked and Kerbel, 2007). Laser Doppler flowmetry demonstrated that vascular shutdown occurred very rapidly, which cannot be attributed to the death of endothelial cells and thus confirm the results of our *in vitro* study on adherent endothelial cell monolayer (Kanthou *et al*, 2006). Specifically, we demonstrated that the electroporation-induced vascular effects can be attributed to the rapid disruption of microfilament and microtubule cytoskeletal networks that paralleled an increase in endothelial monolayer permeability (Kanthou *et al*, 2006). This may lead to increased liquid extravasation and, as a consequence of the increased interstitial fluid pressure, also to the collapse in blood vessel structures. Vasoconstriction due to the effects on smooth muscle cells in tumour blood vessels is less likely, owing to its lack in tumour vessels, as opposed to blood vessels in normal tissues, where this kind of effects was already described (Gehl *et al*, 2002).

Several two- and three-dimensional numerical calculations have demonstrated that an adequate distribution of electric field above the threshold value for electroporation is important for effective electrochemotherapy (Miklavcic *et al*, 1998, 2006). By applying adequate electric pulses with suitable amplitude, most of the tissue positioned between the electrodes should be electroporated. Therefore, stromal cells as well as cell-forming vascular network are also exposed to an electric field that can have electroporative effect. To validate whether endothelial cells in the lining of small tumour vessels are exposed to an electric field that can increase their membrane permeability, a mathematical model was then used. The model predicted that endothelial cells lining the tumour blood vessels are even exposed to ∼40% higher electric field than the surrounding tumour cells. This exposure to a high electric field indicates that tumour endothelial cells are a valid target for electroporation and electrochemotherapy. The application of electric pulses to the tumour, therefore, has a selective, more pronounced, effect on endothelial cells compared to tumour cells. In addition to that, by electrochemotherapy, during electroporation, endothelial cells are exposed to a relatively higher concentration of the chemotherapeutic drug (BLM or cisplatin) compared to tumour cells. Therefore, it can be presumed that electrochemotherapy has a cytotoxic effect on endothelial cells in the treated tumours, which may lead to a prolonged reduction of tumour blood flow. This prolonged reduction in tumour blood flow (vascular disrupting action) has been demonstrated in our previous studies on electrochemotherapy with BLM and cisplatin (Sersa *et al*, 1999a, 2002), as well as in the present study by power Doppler US imaging and Patent blue staining. The measurement of hypoxia in tumours and partial pressure of oxygen do not entirely follow the measurement of reduction of blood flow. The reduction of partial pressure of oxygen was instantaneous but with quick recovery to the pretreatment level within 8 h after application of electric pulses or 48 h after electrochemotherapy of the tumours. These results on tissue oxygenation correlated well with the observed percentage of the pimonidazole-positive area, but not with the reduction of tumour blood flow as demonstrated by power Doppler ultrasonography and Patent blue staining. We demonstrated that the pimonidazole-positive area increased to 35% within 1 h after treatment, with recovery to control values within 12 h after application of electric pulses, whereas tumour blood flow did not recover during the observation period. The reason for that can be that the percentage of pimonidazole-positive area did not increase after electrochemotherapy because in the viable area that remained after therapy, blood flow was restored and the pimonidazole-positive areas were not evaluated in the necrotic areas. Another reason for lower pimonidazole-positive areas can also be ascribed to the fact that the pimonidazole uptake into tumour tissue could be prevented if some parts of the tumours are not perfused at all (owing to the normal fluctuation of tumour blood flow), and this could result in falsely negative areas, despite being hypoxic owing to the treatment. Furthermore, the discrepancy between the oxygenation measurements obtained by EPR oximetry and blood flow reduction measured by power Doppler sonography and Patent blue technique could be due to the differences in the sample volume of the techniques. Specifically, with power Doppler sonography, the whole tumour is assessed, whereas with EPR oximetry, pO_2_ is measured only in the region in contact with the probe that comprises only ∼0.5 mm^3^. As the probes were inserted mainly in the periphery of the tumours, which is part of the tumour that recovers after the treatment with vascular disruption agents, quicker the restoration of partial oxygen pressure compared to tumour blood flow could result from the differences in measurement between the techniques. Nevertheless, the results of partial pressure of oxygen indicated that restoration of oxygen supply in the region of interest after electrochemotherapy is much slower than after electroporation only. This may indicate that many cells undergo cell death, which may take up to 24 h. Furthermore, histological analysis of the tumours indicated that the extent of tumour necrosis after electroporation of tumours paralleled the extent of tumour necrosis after electrochemotherapy up to 24 h after treatment and that only thereafter is there an increase in the extent of tumour necrosis by electrochemotherapy. This may indicate, on a dual effect, the immediate vascular disrupting action of electroporation (increase in vascular permeability due to the disruption of endothelial cytoskeleton and endothelial cell swelling) and delayed effect due to the exposure of these cells to a high concentration of BLM causing endothelial cell death as observed in histological specimens. The first phenomenon is present up to 8–12 h after electroporation of the tissue and is due to disruption of endothelial cell lining in tumour vessels leading to some extent of tumour cell death present up to 24 h, but with quick recovery without significant antitumour effect. The second phenomenon is due to endothelial cell death caused by electrochemotherapy that in addition to the vascular disrupting action of electroporation induces a prolonged shutdown of tumour blood flow and consequently a cascade of tumour cell death in the surrounding tissue that becomes evident at longer times after therapy. Further support for this second phenomenon is the observation that predominantly central tumour necrosis after electrochemotherapy was observed, which is characteristic for vascular disrupting agents.

Electroporation compared to other vascular disrupting agents such as CA-4-P and DMXAA, which have specific effects on tumour blood vessels as opposed to normal blood vessels, is not specific and selectivity is achieved by the local application of electric pulses to the tumours. The vascular disrupting effects of vascular disrupting agents on tumour endothelial cells involve the rapid reorganisation of the actin cytoskeleton, which is mediated by the disruption of the tubulin cytoskeleton for CA-4-P, but not DMXAA (Tozer *et al*, 2005). The signalling pathways associated with CA-4-P and their effects on vascular permeability involve the activation of small GTPase Rho and Rho kinase (Kanthou and Tozer, 2002). The activation of Rho proteins necessitates their translocation and association with membrane components (Bokoch *et al*, 1994). In the case of electroporation, actin as well as microtubule cytoskeleton is disrupted as a consequence of the applied electric field; however, disruption by electroporation appears to uncouple Rho and inactivate Rho kinases. It is likely that electroporation inactivates Rho by preventing its association with the cell membrane (Kanthou *et al*, 2006).

Electroporation, similar to vascular disrupting agents, induces a very rapid and significant reduction in tumour blood flow, which can be detected already within a minute after the application of the treatment. The proposed mechanism for rapid tumour vascular shutdown after treatment with CA-4-P or DMXAA involves the disruption of the cytoskeleton of endothelial cells, leading to cell shape changes and an increase in the permeability of the cell monolayer. This would increase vascular resistance to blood flow. Apoptosis of endothelial cells might also contribute to vascular shutdown. Other contributing factors are platelet activation and serotonin release leading to active vasoconstriction. The protein leakage due to the increased tumour vascular permeability would lead to oedema and an increase in tumour interstitial pressure further leading to vascular collapse. In addition, as the blood flow slows down, red cells stack up causing an increase in viscous resistance and slowing the blood flow by positive mechanisms. The consequence of this is the appearance of central necrosis with a viable peripheral rim of tumour cells, leading to rapid repopulation of tumours and the consequent failure to achieve significant tumour growth delay (Tozer *et al*, 2005). Major vascular disrupting agents that have been tested in clinical phase I studies include CA-4-P, DMXAA, ZD6126, AVE8062 and ABT-751 (Hinnen and Eskens, 2007; Patterson and Rustin, 2007). The combination of vascular disrupting agents with other treatment modalities, such as radiotherapy or chemotherapy, leads to increased antitumour effect of these combined treatments (Tozer *et al*, 2005; Patterson and Rustin, 2007). In the case of electrochemotherapy, electroporation is combined with chemotherapy. This treatment in a single application thus combines the immediate vascular disrupting action of electroporation with delayed endothelial cell death caused presumably by a high chemotherapeutic drug concentration in the cytosol of endothelial cells. Both these effects, in addition to direct tumour cell killing by electrochemotherapy, contribute to the high antitumour effectiveness of electrochemotherapy.

In conclusion, in this study, we provide evidence that electrochemotherapy, in addition to direct cytotoxic effect on tumour cells, has a vascular disrupting action. The vascular disrupting action is dual, namely the effect of application of electric pulses to the tumours that induce a rapid shutdown of tumour blood flow that recovers within 24 h and the continuation of the vascular disrupting effect of electrochemotherapy, predominantly through its cytotoxic effect on endothelial cells.

## Figures and Tables

**Table 1:** Tumour growth after electrochemotherapy with bleomycin

**Group**	***N***	**Tumour doubling time (days; AM±s.e.)**	***P*-value compared to tumour doubling time of control**	**Tumour growth delay (days; AM±s.e.)**	**Cures *n* (%)**
Control	16	1.2±0.06			0
Bleomycin^a^	16	1.2±0.04	NS	0.0	0
Electric pulses^b^	16	1.8±0.02	NS	0.6±0.02	0
Electrochemotherapy^c^	16	23.7±4.6	*P*<0.05	22.5±4.6	6 (38)

NS=nonsignificant.

a Bleomycin injected intravenously (1 mg kg^−1^).

b Application of electric pulses to the tumour (8 pulses, 1040 V, 100 *μ*s, 1 Hz).

c Electrochemotherapy: application of electric pulses to the tumour 3 min after intravenous injection of bleomycin.

**Table 2:** Partial pressure of oxygen (pO_2_) measured by EPR oximetry in SA-1 tumours and subcutis before the treatment of tumours and 2 h after treatment by electric pulses, bleomycin and electrochemotherapy

	**Pretreatment values**	**Bleomycin^a^**	**Electric pulses^b^**	**Electrochemotherapy^c^**
**Tissue/therapy**	**(mmHg, AM±s.e.)**	**(mmHg, AM±s.e.)**	**(mmHg, AM±s.e.)**	**(mmHg, AM±s.e.)**
Subcutis	8.37±0.05	7.35±0.09^d^	7.66±0.08^d^	5.90±0.17^d^
Tumour periphery	7.33±0.09	4.93±0.19^d^	2.16±0.07^d^	2.11±0.13^d^
Tumour centre	5.35±0.05	4.58±0.07^d^	2.68±0.14^d^	1.39±0.12^d^

EPR=electron paramagnetic resonance.

a Bleomycin injected intravenously (1 mg kg^−1^).

b Application of electric pulses to the tumour (8 pulses, 1040 V, 100 *μ*s, 1 Hz).

c Electrochemotherapy: application of electric pulses to the tumour 3 min after intravenous injection of bleomycin.

d *P*-values (<0.05) for different treatments *vs* normal values.
